# Spatial clustering of non-transported cardiac decedents: the results of a point pattern analysis and an inquiry into social environmental correlates

**DOI:** 10.1186/1476-072X-10-46

**Published:** 2011-07-28

**Authors:** Elizabeth Barnett Pathak, Steven Reader, Jean Paul Tanner, Michele L Casper

**Affiliations:** 1 Department of Epidemiology and Biostatistics College of Public Health, University of South Florida 13201 Bruce B. Downs Blvd. MDC 56 Tampa FL 33612 USA; 2 Department of Geography, Environment, and Planning University of South Florida 4202 E Fowler Ave, NES107 Tampa FL 33629-5250 USA; 3 Division for Heart Disease and Stroke Prevention National Center for Chronic Disease Prevention and Health Promotion Centers for Disease Control and Prevention 4770 Buford Highway MS-K-47 Atlanta, GA 30341-3724 USA

## Abstract

**Background:**

People who die from heart disease at home before any attempt at transport has been made may represent missed opportunities for life-saving medical intervention. In this study, we undertook a point-pattern spatial analysis of heart disease deaths occurring before transport in a large metropolitan area to determine whether there was spatial clustering of non-transported decedents and whether there were significant differences between the clusters of non-transported cardiac decedents and the clusters of transported cardiac decedents in terms of average travel distances to nearest hospital and area socioeconomic characteristics. These analyses were adjusted for individual predictors of transport status.

**Methods:**

We obtained transport status from the *place of death *variable on the death certificate. We geocoded heart disease decedents to residential street addresses using a rigorous, multistep process with 97% success. Our final study population consisted of 11,485 adults aged 25-74 years who resided in a large metropolitan area in west-central Florida and died from heart disease during 1998-2002. We conducted a kernel density analysis to identify clusters of the residential locations of cardiac decedents where there was a statistically significant excess probability of being either transported or not transported prior to death; we controlled for individual-level covariates using logistic regression-derived probability estimates.

**Results:**

The majority of heart disease decedents were married (53.4%), male (66.4%), white (85.6%), and aged 65-74 years at the time of death (54.7%), and a slight majority were transported prior to death (57.7%). After adjustment for individual predictors, 21 geographic clusters of non-transported heart disease decedents were observed. Contrary to our hypothesis, clusters of non-transported decedents were slightly closer to hospitals than clusters of transported decedents. The social environmental characteristics of clusters varied in the expected direction, with lower socioeconomic and household resources in the clusters of non-transported heart disease deaths.

**Conclusions:**

These results suggest that in this large metropolitan area unfavorable household and neighborhood resources played a larger role than distance to hospital with regard to transport status of cardiac patients; more research is needed in different geographic areas of the United States and in other industrialized nations.

## Background

Geographic studies of chronic disease outcomes have often relied on predefined geographic units (e.g., they have frequently used rates for county or census tracts) [1-3]. However, point-pattern analyses can provide a much more nuanced understanding of the spatial patterns and geographic determinants of chronic disease outcomes, especially for those cardiovascular diseases in which timely access to definitive care has been shown to be an important determinant of outcomes. Diseases of the heart, a common and serious family of specific disorders, have a complex etiology which includes individual-level biomedical, behavioral, and psychosocial causes [4], as well as community and structural determinants [5].

In recent years, medical advances and new recommendations for treatment from the American Heart Association and American College of Cardiology [6,7] have dramatically improved survival rates for heart disease, the leading cause of death in the United States. In the event of a heart attack or other acute cardiac event, people with heart disease have very good chances of survival if they are rapidly transported to a hospital and receive medical care that includes interventions according to guidelines [6]. Conversely, those who die from heart disease at home prior to any attempt at transport may represent missed opportunities for life-saving medical intervention. Previous research has suggested that both individual-level (e.g., being unmarried) [8,9] and area-level (e.g., living in the Western region of the U.S.) [8] characteristics may be important in determining whether someone receives transport prior to death. However, these earlier studies examined relatively large geographic units and did not examine point patterns of mortality from heart disease.

In this study, we undertook a point-pattern spatial analysis of heart disease deaths in a large metropolitan area in Florida to determine whether (1) spatial clustering of non-transported and/or transported deaths occurred and (2) there were significant differences between cluster types in average hospital travel distances and area socioeconomic characteristics, after adjustment for individual predictors of transport status. We analyzed heart disease mortality data from 1998-2002 for adults aged 25-74 years old, and hypothesized that clusters of non-transported heart disease decedents would be found in residential areas with relatively fewer socioeconomic resources and further distances to an acute-care hospital than we would find in areas where there were clusters of transported deaths.

## Methods

### Study Population

The geographic area of our study was the Tampa-St.Petersburg-Clearwater Metropolitan Statistical Area (MSA) in central Florida, hereafter referred to as the "Tampa MSA." Our study population consisted of adults aged 25-74 years old who were residents of the Tampa MSA, and who died from heart disease during 1998-2002. We ascertained cardiac decedent status from death certificate data files obtained from the State of Florida Vital Statistics Office. A death from heart disease was defined as any death for which the underlying cause was *diseases of the heart *(ICD-9 [International Classification of Diseases, 9^th ^Revision] codes 390-426; ICD-10 [International Classification of Diseases, 10^th ^Revision] codes I00-I09, I11, I13, I20-I51), or *symptoms, signs, and ill-defined conditions *(ICD-9 codes 780-799; ICD-10 codes R96-R99) [10-12]. In this final category, however, we excluded any deaths for which the underlying cause was *senility *(ICD 9 code 797; ICD 10 code R54), as these deaths were unlikely to be cardiac related.

There were 12,952 heart disease deaths among residents aged 25-74 years old in the Tampa MSA during 1998-2002. We excluded 412 (3.2%) decedents whose addresses could not be geocoded, and 250 (1.9%) decedents who resided in nursing homes at the time of their deaths. The study population was comprised of 12,290 non-institutionalized heart disease decedents, prior to further geographic exclusions as explained below. We obtained study approvals from the Florida Department of Health and the University of South Florida Institutional Review Board.

### Geographic Variables

We used residential addresses as recorded on the death certificates to geocode each death from heart disease. We used a rigorous, multistep process that resulted in 97% of all decedents being successfully geocoded to a specific street address. We started with the geocoding utility built into the ESRI ArcGIS software package (ESRI, Redlands, California), which we supplemented by extensive manual checking and case-by-case hand geocoding for all cases where the address on the death certificate did not exactly match the ArcGIS street database. Problems we frequently encountered were incorrect zip codes, misspelled street names, and house numbers which were not in range. We used the ESRI ArcGIS street database as our reference for geocoding, supplemented by detailed zip-code and street maps of the entire metropolitan area. None of the decedents were geocoded to zipode centroids or other approximate locations.

Using multiple local data sources, we created a comprehensive list of acute-care general hospitals that were active during 1998-2002 in the 4-county Tampa MSA. Specialty and long-term care hospitals without general emergency departments were excluded from our analyses. Published hospital addresses were used to geocode and map the 29 hospitals that were included. After geocoding was completed a road network-based route analysis was undertaken to calculate driving distances (in miles) from each decedent's home to the nearest acute-care hospital.

**Figure 1:**
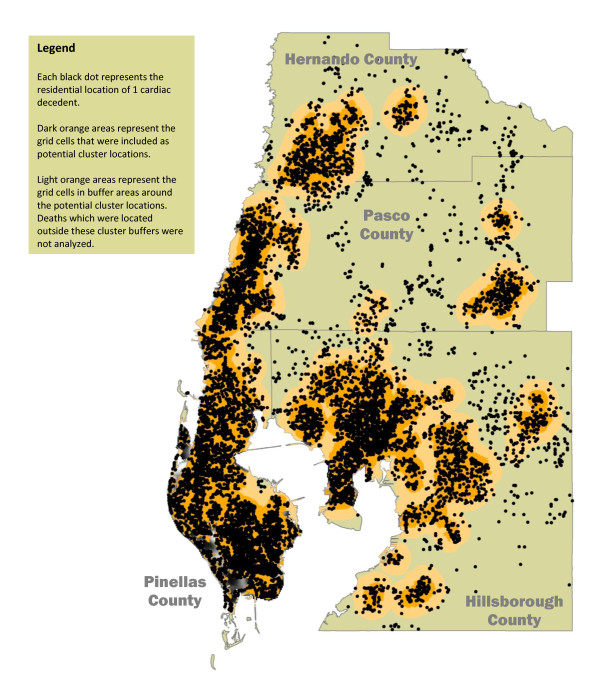
**Spatial Distribution of Cardiac Decedents Aged 25-74 Years Across the Tampa Metropolitan Statistical Area in 1998-2002**.

A map of the Tampa MSA along with the spatial distribution of cardiac decedents by residential location is shown in Figure 1. This ethnically and geographically diverse metropolitan area of over 2 million people encompasses 2,600 square miles of central cities, suburbs, small towns, and rural farms. As expected, decedents were concentrated in the densely populated urban areas of the MSA. As described in the section on statistical analysis below, we imposed a spatial filter on the number of deaths that resulted in most of the land area in Hernando, Pasco, and Hillsborough counties (i.e., rural farmland) being excluded from the statistical analyses. In all, 805 deaths were excluded, resulting in a final study population of 11,485 cardiac decedents.

### Outcome Variable

Our outcome in this study was no transport prior to cardiac death; we obtained information on transport status for each decedent from the *place of death *variable on the death certificate. We categorized a cardiac death as occurring with no transport if place of death was reported as either (1) at home; or (2) in another nonhospital location in the community. Deaths coded as occurring during or after transport had place of death recorded as one of the following: (1) dead on arrival (at hospital); (2) emergency room/outpatient; (3) hospital: inpatient; (4) hospital: status unknown (inpatient vs. outpatient status). Therefore, for each decedent the outcome variable was dichotomous: non-transported or transported. We excluded deaths among nursing home residents because the determinants of their transport status were likely to be qualitatively different from those of decedents who had lived in the community [Anic G, Pathak EB, Tanner JP, Casper ML, Branch LG: Transport to hospital prior to heart disease death among nursing home residents: the role of nursing home and individual demographic characteristics, submitted].

### Spatial Statistical Analyses

We conducted a kernel density analysis [13] to identify clusters of the residential locations of cardiac decedents with a statistically significant excess probability of being transported (versus not being transported) prior to death. A kernel density analysis essentially creates a relative measure of point-pattern intensity between a set of "cases" (in this case non-transported deaths) and a set of "controls" (in this case transported deaths) for each of a set of grid cells that then form a continuous grid surface across the study area. Therefore, in the context of our study, the kernel density analysis produced a type of spatial smoothing in which a cluster test for a very small geographic area (in our study a grid cell was 200m^2^) was conducted using all the neighboring cases within a specified radius (2 km). We also used a spatial filter of ≥30 deaths to limit the cluster testing to grid cells with a sufficient numbers of deaths within the radius of 2 km. The spatial filter was necessary because some regions in our study area were rural and sparsely populated.

We first calculated the kernel density surface of the 4,863 non-transported heart disease deaths using the 200m^2 ^grid cells. For each grid cell, that analysis resulted in a distance-weighted count of non-transported deaths. Next, we created multiple simulated kernel density surfaces based on random Monte Carlo samples. We accomplished this by randomly selecting 4,863 deaths from the total group of 11,485 without regard to their transport status. This process was repeated 999 times, resulting in 999 simulated kernel density surfaces that were then compared with the kernel density surface of the non-transported deaths. For each grid cell, where the kernel density value (for the non-transported decedents) was higher than the simulated values for >990 of the simulations (i.e., in the top 1% to achieve a 99% significance level), that grid cell was identified as part of a cluster of non-transported cardiac deaths. Similarly, where the kernel density value (for the transported decedents) was lower than the simulated values for >990 of the simulations, that grid cell was identified as part of a cluster of *transported*cardiac deaths.

To further refine our analyses, we controlled for individual-level covariates by using the results of logistic regression modeling to adjust the kernel density analysis. Specifically, using the data for individual decedents, we modeled the probability of a decedent not being transported as a function of age, gender, race/ethnicity, and marital status. These individual-level characteristics have all been shown to be important predictors of transport status for cardiac decedents [8,9]. The individual probabilities generated by the logistic model were then used to weight the selection of deaths for each of the 999 Monte Carlo simulations. As a result, decedents who were younger, male, white, and/or unmarried (all factors that increase the likelihood of no transport) were more likely to be selected for any given simulation. Therefore, this approach was conservative, and allowed us to determine whether clusters of transported and non-transported decedents existed/persisted after adjustment for known individual predictors of transport status.

### Analysis of the Social Environmental Characteristics of Clusters

After clusters of transported and non-transported deaths adjusted for individual-level correlates were identified, we used data from the U.S. Census of Population and Housing for 2000 (the midpoint of our study period) to describe the social environmental characteristics of the clusters. For this analysis, the cluster boundaries were defined by the 2 km radius buffer around contributing grid cells. Using the ESRI Arc-GIS software, we first overlaid the cluster boundaries onto the boundaries of the census blocks. The boundaries of the census block groups were intersected with the boundaries of the clusters we had identified to determine which census block groups fell within each cluster. Some clusters contained multiple block groups, including partial block groups. We examined 8 household and neighborhood characteristics that we hypothesized would affect the likelihood of transport status prior to a heart disease death: median income, percent living with family, percent living alone, percent living in group quarters, percent rental units, percent single-person households, percent without telephone service, and percent with no vehicle available. For each variable a value for the cluster was calculated as a weighted average of the complete and partial census block groups that fell within the cluster, based on the block group population that fell within the cluster.

## Results

Characteristics of the study decedents are shown in Table 1. The majority were married (52.7%), male (66.4%), white (85.0%), and aged 65-74 years at the time of death (54.9%). Overall, a modest majority of decedents were transported prior to death (57.7%). Among those who were transported, 34.4% died in the hospital emergency department or were dead on arrival at the hospital. Of the 4,863 decedents who were not transported prior to death, 77.9% died at home.

**Table 1:** Characteristics of Cardiac Decedents Aged 25-74 (n = 11,485), Tampa MSA, 1998-2002

Decedent Characteristics	Percent (Number)
**Year**	

1998	21.7 ( 2,491)

1999	20.5 ( 2,357)

2000	20.4 ( 2,347)

2001	19.0 ( 2,181)

2002	18.4 ( 2,109)

**Age (years)**	

25-44	7.1 ( 816)

45-64	38.0 ( 4,360)

65-74	54.9 ( 6,309)

**Gender**	

Women	33.6 ( 3,862)

Men	66.4 ( 7,623)

**Race/Ethnicity**	

White	85.0 ( 9,763)

Black	11.1 ( 1,270)

Hispanic	3.9 ( 452)

**Marital Status**	

Married	52.7 ( 6,051)

Widowed	15.0 ( 1,724)

Divorced	21.6 ( 2,485)

Single	9.1 ( 1,047)

Unknown	1.6 ( 178)

**Underlying Cause of Death**	

Diseases of the Heart	96.1 (11,034)

Ill-Defined Symptoms/Signs	3.9 ( 451)

**Transport Status**	

Not Transported	42.3 ( 4,863)

Transported	57.7 ( 6,622)

**Place of Death for** **Non-Transported Decedents**	

Home	77.9 (3,789)

Other (not Hospital)	22.1 (1,074)

**Place of Death for** **Transported Decedents**	

Inpatient	65.6 (4,346)

Emergency/Outpatient/DOA	34.4 (2,276)

DOA: Dead on arrival.

**Figure 2:**
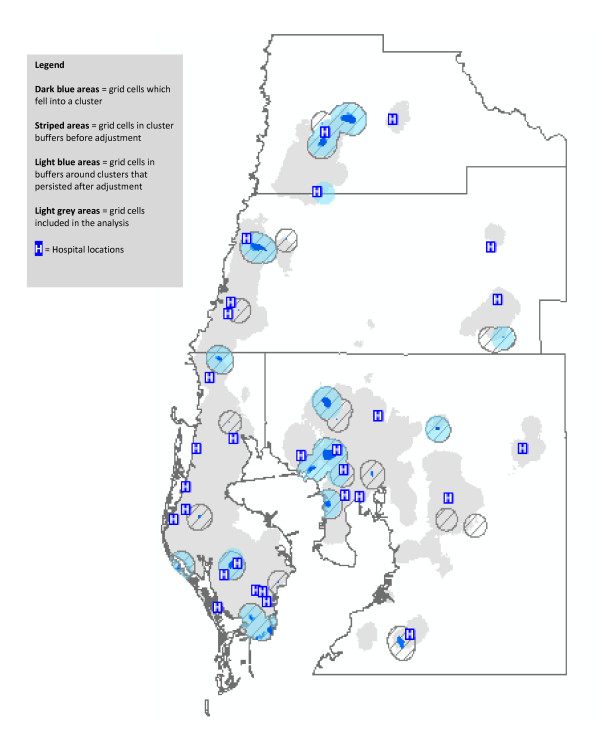
**Clusters of Transported Heart Disease Decedents, Before and After Adjustment for Individual Predictors of Transport Status**.

The results of the cluster analyses for transported cardiac deaths are shown in Figure 2. The light grey shading represents the grid cells with adequate numbers of deaths to be included in the spatial analyses. The dark blue areas show clusters of transported cardiac deaths, both before and after adjustment for individual predictors of transport status. The striped areas represent the unadjusted cluster buffers. Cluster buffers identify the radius of 2 km that contributed cardiac deaths to the clusters. The light blue areas identify the cluster buffers after adjusting for individual predictors of transport status. As can be seen from the map, some of the cluster buffers overlap each other. This is an expected consequence of the kernel density analysis.

Before adjustment, the kernel density analysis identified 29 separate clusters of transported deaths. After adjustment for individual predictors, 12 of these clusters disappeared, which means that the apparent spatial clustering in those 12 areas was the result of individual characteristics of the decedents who lived there. Of note, there was one new cluster which appeared after adjustment for individual characteristics. Most of the clusters of transported deaths which persisted after adjustment were located in close proximity to a hospital, although 3 of the 17 clusters were not.

**Figure 3:**
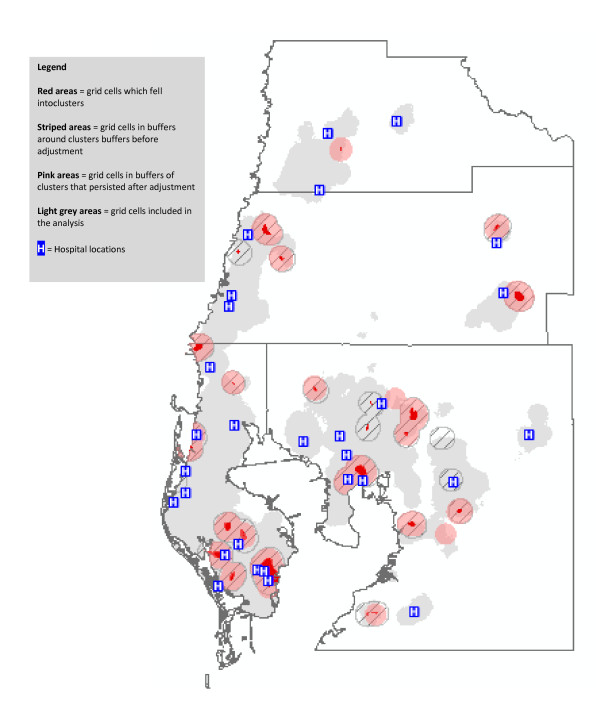
**Clusters of Non-Transported Heart Disease Decedents, Before and After Adjustment for Individual Predictors of Transport Status**.

Figure 3 depicts clusters of non-transported heart disease deaths. The unadjusted kernel density analysis revealed 28 clusters (red clusters surrounded by striped buffers), of which 21 remained after adjustment for individual factors (the 21 are shown as red clusters with pink cluster buffers). Three new and very small clusters appeared after adjustment (these clusters do not have striped buffers). Our hypothesis that clusters of non-transported deaths would occur farther away from acute-care hospitals was not supported: most of these clusters were in close proximity to a hospital.

**Figure 4:**
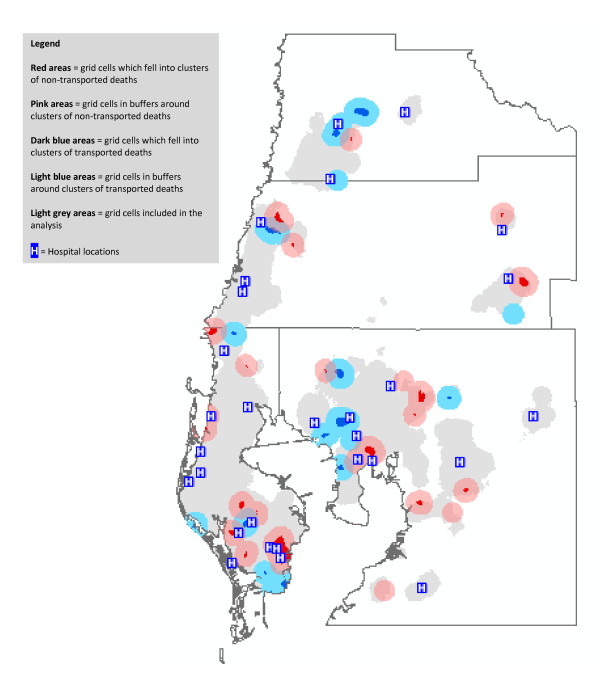
**Clusters of Non-Transported vs. Transported Heart Disease Decedents, After Adjustment for Individual Predictors of Transport Status**.

Figure 4 shows the locations of clusters resulting from the adjusted analyses for both transported and non-transported deaths. In general, clusters of non-transported deaths appeared to be closer to hospitals (or equidistant) than were clusters of transported deaths, with a few exceptions seen to this general pattern, notably in eastern Hillsborough County. We quantified this apparent effect by calculating the distributions of travel distances (by road) for decedents in transported clusters versus non-transported clusters (we included decedents in the respective cluster buffers in our calculations.) The median distance to the nearest hospital in the non-transported clusters was 2.3 miles (range, 0.1 - 11.8 miles), versus a median distance of 2.6 miles (range, 0.4 - 14.4 miles) for decedents in the transported clusters. These differences are not great, and most decedents lived within relatively close to an acute-care hospital.

Finally, we examined whether social environmental characteristics of the non-transported clusters differed significantly from those of the transported clusters after controlling for individual-level predictors of transport status (Table 2). The population residing in the non-transported clusters were more likely to have an income below the poverty level (14% vs. 11%), live alone (14% vs. 12%), live in a rental unit (35% vs. 30%), live in a household with no phone service (3% vs. 2%), and live in a household with no vehicle available (12% vs. 8%). Overall, the socioeconomic resources of all of these areas were moderately high, with a median household income of $38,187 in the non-transported clusters (versus $40,515 in the transported cluster), and 76% of all persons living with family (versus 80% in the transported cluster).

**Table 2:** Social Environmental Characteristics of Census Blocks Underlying the Clusters of Transported vs. Non-Transported Decedents

Social Environmental Characteristics of Census Blocks Underlying Clusters	Adjusted* Clusters of Transported Deaths (Tot. Pop. = 284,351)	Adjusted* Clusters of Non- Transported Deaths (Tot. Pop. = 440,003)
Median household income	$40,515	$38,187

% of persons below poverty income	11	14

% of persons living with family	80	76

% of persons living alone	12	14

% of persons living in group quarters	1	3

% of occupied housing units that were rented	30	35

% of households with 1 person	30	33

% of households with no telephone service	2	3

% of households with no vehicle	8	12

*Clusters were defined using estimates adjusted for individual decedents' age, gender, race/ethnicity, and marital status. See Methods for details.

## Discussion

Lack of transport for acute cardiac events can have several causes, including delays at the patient level in seeking treatment [14]; barriers to transportation at the household level; and geographic location [8], lack of emergency medical services [15], and distance from a hospital at the community level [16]. In our investigation, contrary to what we hypothesized, we found that distance to a hospital was not predictive of transport status, with most clusters of non-transported decedents located near a hospital and, indeed, slightly closer to a hospital than clusters of transported decedents. In contrast, social environmental characteristics of clusters varied in the expected direction, with clusters of non-transported decedents having, on average, lower socioeconomic and household resources. Our entire 4-county geographic study area is well-served by paramedic-based public EMS agencies, so it is unlikely that spatial variability in EMS availability could account for our findings.

Previous research on the spatial epidemiology of heart disease has focused mostly on chloropleth mapping and conventional statistical analyses of small-area disparities in heart disease death rates in both the U.S. [1,2,17] and the United Kingdom [18]. These studies have found substantial geographic variation in death rates from heart disease, with areas having high death rates likely to have fewer socioeconomic and medical care resources. An interesting study conducted in Rochester, New York, used emergency medical services (EMS) data and geographic information system (GIS) methods to identify clusters of out-of-hospital cases of cardiac arrest. That study found that neighborhood clusters of cardiac arrest patients were more likely in areas where residents had lower educational attainment and lower household incomes than the general population of Rochester [19]. To our knowledge, our study is the first to examine geographic clustering of non-transported heart disease decedents.

### Strengths and Limitations

Important strengths of our study include the availability of comprehensive surveillance data on cardiac decedents, precise street-level geocoding, the GIS route analysis of the travel distance to the nearest hospital for each decedent, and the rigorous kernel density analysis we used to identify clusters. The inferential testing of the clusters using probability weights estimated by logistic regression is a method first introduced into the public health literature in a study of low birth weight incidence by one of this paper's authors [20], and has the potential for wide application in geographical epidemiology.

One of the limitations of this study is the potential for misclassification of cause of death, as is true in any study that relies on death certificates. We chose a relatively broad definition of heart disease to minimize this problem, and we included deaths coded to ill-defined symptoms and signs (excluding senility) in order to improve our capture of out-of-hospital sudden cardiac deaths, for which the coding of cause of death is often vague. The cause-of-death category *ill-defined and unknown causes *(ID) is used by physicians, coroners, and medical examiners when there is insufficient post-mortem evidence to support assigning a specific disease as cause of death [10]. It should be noted that deaths resulting from any kind of injury or external cause are not coded to ID, and in our study only 3.9% of all deaths were coded to ID. Earlier research on sudden cardiac arrest fatalities indicated that these deaths were often coded to ID on the death certificate [11]. Furthermore, previous studies found that heart disease death rates for which ID deaths had been excluded from the numerator were significantly underestimated in some populations [11]. A study of MONICA (Multinational Monitoring of Trends and Determinants in Cardiovascular Disease) data from Belgium found that approximately 5% of "definite" or "possible" cases of acute myocardial infarction had been coded to ID on the death certificate [12].

A second limitation of our study was a methodological problem that is well described in the geographical literature and affected our results as well. This is the dependent relationship between the geographical scale of analysis and the size and location of clusters. In essence, the detection of clusters is a scale-dependent process, and results may vary if different parameters are incorporated into the kernel density algorithms. A third limitation is the potential for geographic bias resulting from a geocoding failure for a small subset of deaths. However, based on the findings of a methodological assessment of the impact of geocoding failure on detection of disease clusters, such bias is unlikely in our study [21]. A fourth limitation is that the method of cluster detection used here is subject to the multiple testing problem since the individual statistical tests for grid cells that share observations for those tests are not independent. This is a well known aspect of this type of analysis, and is partly offset in this study by the conservative significance levels used. Finally, the data on individual risk factors and on the socioeconomic characteristics of the areas studied was not as complete as we would have liked. Adjustment for a broader range of individual factors might have resulted in a different pattern of clustering of non-transported cardiac decedents. There is a need for future research that can draw on linked data sources at both the individual and social environmental levels to improve our understanding of the barriers to rapid medical care for heart disease patients.

## Conclusion

Our study found statistically significant geographic clustering of non-transported cardiac decedents aged 25-74 years at the time of death in a large Florida MSA. The social environmental characteristics of clusters varied in the expected direction, with lower socioeconomic and household resources in the clusters of non-transported deaths. Distance from hospital did not explain the locations of clusters. These results suggest that in this large metropolitan area, household and neighborhood resources may create greater barriers to transport of cardiac patients than hospital distance.

## Competing interests

The authors declare that they have no competing interests.

## Authors' contributions

EBP, SR, and MC conceived of and designed the study. EBP, SR, and JPT conducted the statistical and spatial analyses. EBP and SR drafted the manuscript. All authors edited and approved the final manuscript.
